# Hypertrophic cardiomyopathy with anteriorly directed mitral regurgitation is a red flag for concomitant pathology: a case report

**DOI:** 10.1093/ehjcr/ytae121

**Published:** 2024-03-08

**Authors:** Vincenzo Somma, Jaishankar Raman, Leigh Fitzpatrick, David Prior, Elizabeth Paratz

**Affiliations:** Department of Cardiology, St Vincent’s Hospital Melbourne, 41 Victoria Parade, Fitzroy, VIC 3065, Australia; Department of Cardiothoracic Surgery, St Vincent’s Hospital Melbourne, 41 Victoria Parade, Fitzroy, VIC 3065, Australia; Department of Intensive Care, Albury-Wodonga Health, 201 Borella Road, East Albury, NSW 2640, Australia; Department of Cardiology, St Vincent’s Hospital Melbourne, 41 Victoria Parade, Fitzroy, VIC 3065, Australia; Department of Cardiology, Albury-Wodonga Health, Vermont St, Wodonga, VIC 3690, Australia; Faculty of Medicine, Dentistry and Health Sciences, Melbourne University, Grattan St, Parkville, VIC 3000, Australia; Department of Cardiology, St Vincent’s Hospital Melbourne, 41 Victoria Parade, Fitzroy, VIC 3065, Australia; Faculty of Medicine, Dentistry and Health Sciences, Melbourne University, Grattan St, Parkville, VIC 3000, Australia; HEART Research Lab, St Vincent’s Institute of Medical Research, 9 Princes St Fitzroy, Fitzroy, VIC 3065, Australia; Department of Sports Cardiology, Baker Heart and Diabetes Institute, 75 Commercial Rd, Prahran, VIC 3181, Australia

**Keywords:** Systolic anterior motion, Hypertrophic cardiomyopathy, Diagnostic challenges, Mitral valve, Case report

## Abstract

**Background:**

Hypertrophic cardiomyopathy (HCM) is often linked to systolic anterior motion (SAM) of the mitral valve, typically resulting in a posteriorly directed mitral regurgitation (MR) jet. An anteriorly directed MR jet suggests additional mitral valve pathology that may not be resolved by myectomy alone.

**Case summary:**

A 58-year-old construction worker with no significant medical history experienced a syncopal event and was admitted to the emergency department with acute pulmonary oedema. A systolic murmur was investigated with a trans-thoracic echocardiogram that revealed severe MR with an unusual anteriorly directed MR jet and a possible flail segment of the posterior leaflet. This finding was further characterized with a trans-oesophageal echocardiogram that revealed severe asymmetric septal hypertrophy with SAM of the mitral valve, severe mitral regurgitation into a dilated left atrium with pulmonary vein flow reversal not caused by HCM-associated SAM, and a markedly abnormal mitral valve with flail and prolapse. The patient underwent successful cardiac surgery, including mitral valve repair and septal myectomy. The patient’s recovery was uneventful, allowing for a return to work within a month post-surgery.

**Discussion:**

The anteriorly directed MR jet served as a red flag, leading to the discovery of an independent mitral valve pathology that required surgical intervention beyond the expected treatment for SAM-associated HCM. This case highlights the complexity of assessing MR in patients with HCM and underscores the importance of characterizing MR jet direction in diagnosing additional mitral valve diseases.

**Learning points**

- Systolic anterior motion of the mitral valve is a classic finding in hypertrophic cardiomyopathy with obstruction, however the resultant mitral regurgitation jet should be posteriorly directed.
- Anteriorly directed mitral regurgitation in hypertrophic cardiomyopathy should be considered a red flag for the presence of additional mitral valve pathology unrelated to systolic anterior motion of the mitral valve.

## Introduction

Hypertrophic cardiomyopathy (HCM) is classically associated with systolic anterior motion (SAM) of the mitral valve. Colour Doppler echocardiographic assessment can identify SAM-induced MR by a regurgitant jet with a typical posterior orientation.^1^ A jet that is not directed posteriorly implies the possibility of intrinsic mitral valve disease, unrelated to SAM, and the associated MR may not be rectified by myectomy alone. Identification of an anteriorly directed MR jet should act as a red flag to the reviewing physician that MR is not due to SAM alone and investigation for additional causes should be undertaken.^2,3^

## Summary figure

**Figure:**
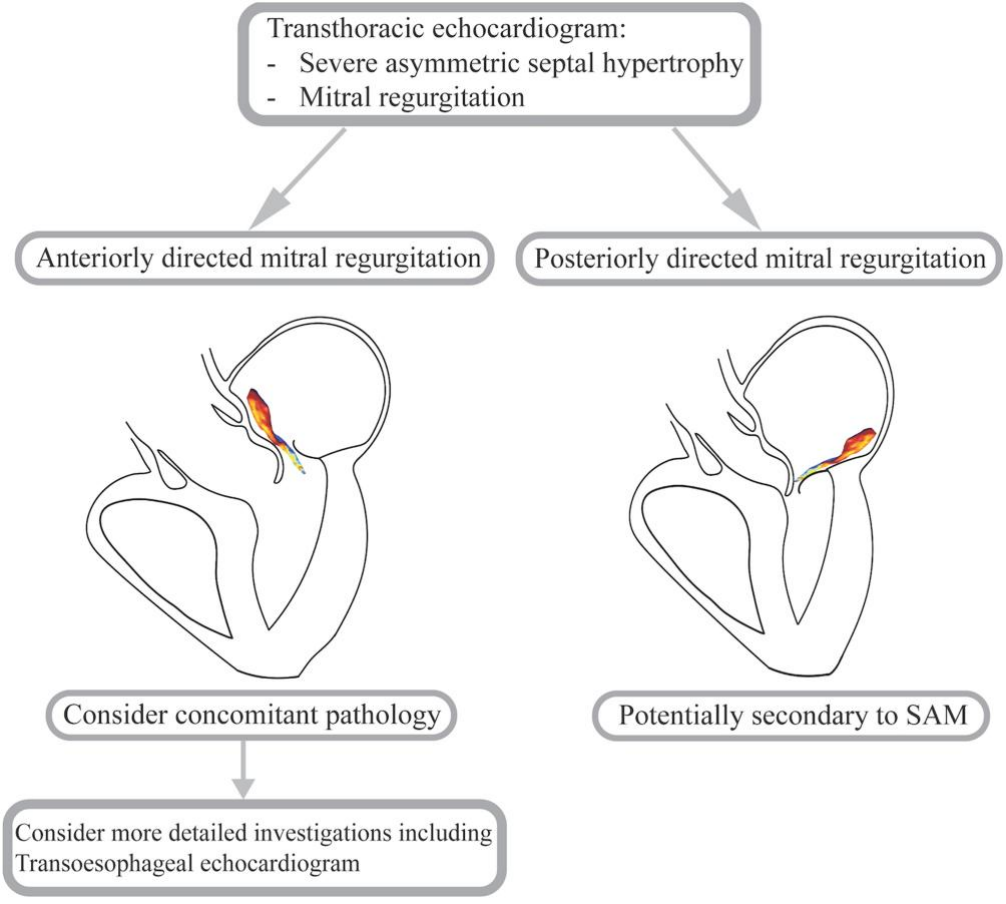


## Case report

A 58-year-old man was brought to a regional emergency department after an unwitnessed syncopal event. The patient had no known medical history other than a previous inguinal hernia repair.

On arrival to the emergency department, the patient initially demonstrated signs of acute pulmonary oedema with bilateral infiltrates on chest X-ray and requiring oxygen therapy at 4 L/min (via nasal prongs) to maintain oxygen saturations of 94%. Serial troponin-I levels were normal, and electrocardiogram demonstrated a left bundle branch block with first degree AV block and voltage criteria fulfilled for left ventricular hypertrophy. On examination, a systolic murmur was heard. The patient was admitted to the ward, and intravenous diuresis was commenced.

**Figure 1:**
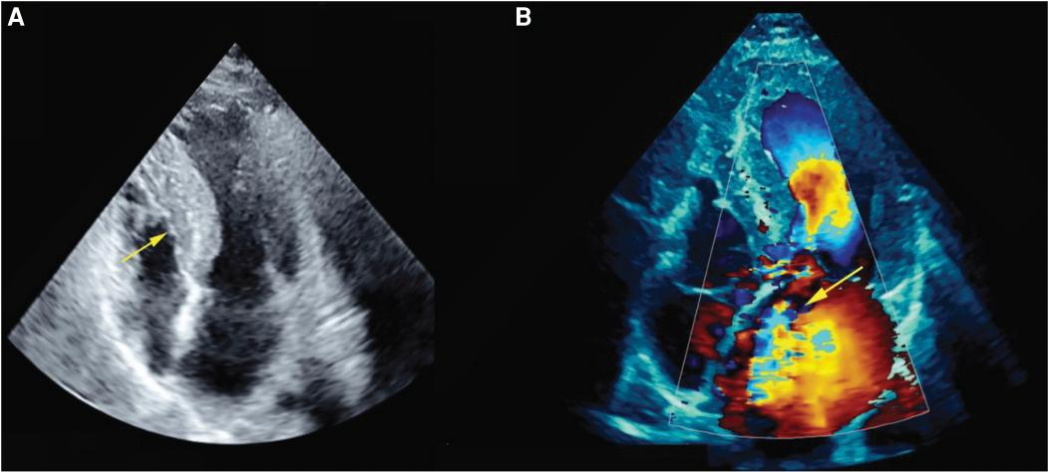
Trans-thoracic echocardiography apical four-chamber view demonstrating asymmetric thickened ventricular septum (yellow arrow) (*A*). Initial trans-thoracic echocardiography apical four-chamber view Doppler demonstrating anteriorly directed jet of mitral regurgitation (*B*).

On Day 2 of admission, the patient deteriorated, developing cardiogenic shock and respiratory compromise with a blood pressure of 70/50 mmHg, heart rate of 140 beats/min (atrial fibrillation), and severe hypoxia requiring intubation. Bedside cardiac ultrasound revealed asymmetric septal hypertrophy (maximal septal thickness = 19 mm) and mitral valve systolic SAM consistent with a new diagnosis of hypertrophic obstructive cardiomyopathy. Anteriorly directed MR was noted (*Figure 1*). The patient was commenced on beta-blocker (metoprolol 37.5 mg b.i.d.) therapy to control ventricular rate and reduce left ventricular outflow tract obstruction (LVOTO). There was persistent pulmonary congestion, and a formal trans-thoracic echocardiogram (TTE) revealed severe anteriorly directed MR and possible flail segment of P2/3 scallops of the posterior leaflet (*Figure 1*).

**Figure 2:**
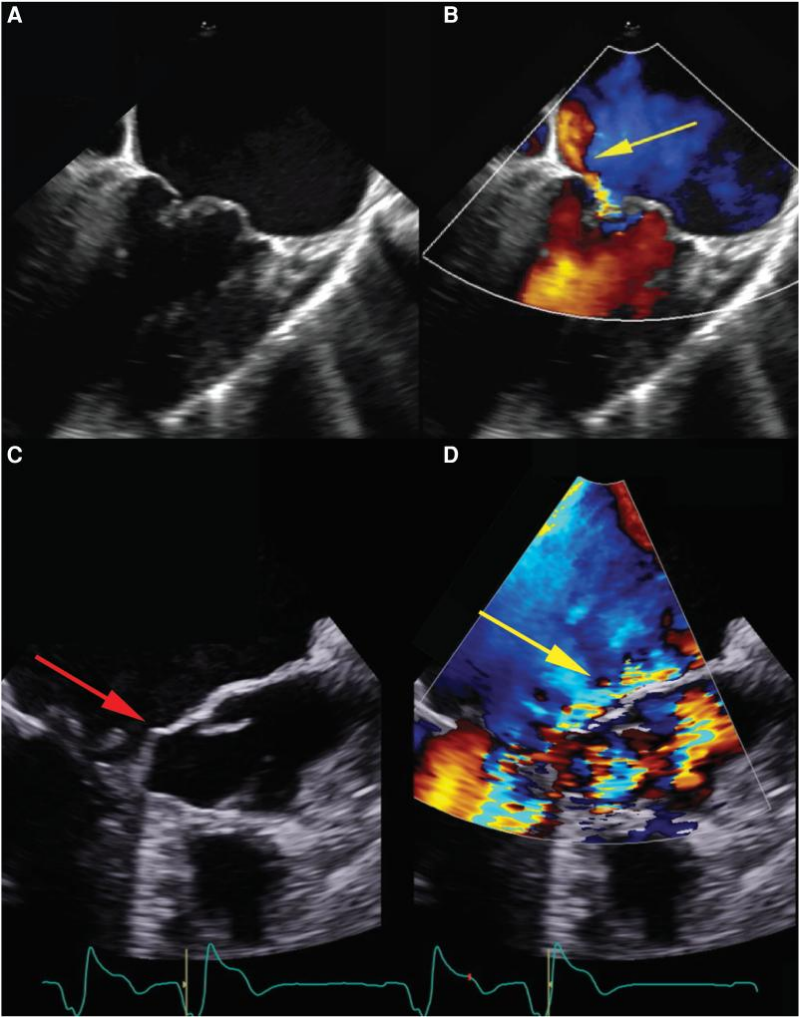
Trans-oesophageal echocardiography, mid-oesophageal mitral bi-commissural view demonstrating mitral valve P2/3 posterior leaflet prolapse (*A*) as the cause of anteriorly directed SAM (arrow) (*B*). SAM of the mitral valve (arrow) (*C*) and anteriorly directed mitral regurgitation (arrow) (*D*).

The patient underwent trans-oesophageal echocardiogram (TOE) (*Figure 2*), demonstrating severe asymmetric septal hypertrophy with severe dynamic LVOTO (peak resting gradient 100 mmHg) due to SAM of the mitral valve (Supplementary material online, *Videos S1* and *S2*). Severe MR into a severely dilated left atrium was identified, with systolic flow reversal in 3 of 4 pulmonary veins, but was determined not to be due to the HCM-associated SAM. There was a markedly abnormal mitral valve with a flail middle scallop (P2) of the posterior leaflet and a minor region of prolapse in the medial P3 scallop creating severe anteromedial MR (Supplementary material online, *Video S3*). Biventricular systolic function was normal. Given these findings the patient was transferred to a tertiary centre for cardiac surgery.

The patient was transferred intubated, with stable respiratory parameters. Haemodynamically, the patient had inotropic requirements of norepinephrine at 5 μg/min and vasopressin at 3 units/min to maintain a heart rate of 55 beats/min, blood pressure of 115/60 mmHg, and a central venous pressure of 11–14 mmHg with good urine output. He was in intermittent atrial fibrillation. A coronary angiogram revealed normal coronary arteries, an elevated left ventricular end-diastolic pressure of 29 mmHg (normal < 12 mmHg) and peak left ventricular outflow tract (LVOT) gradient of 60 mmHg.

**Figure 3:**
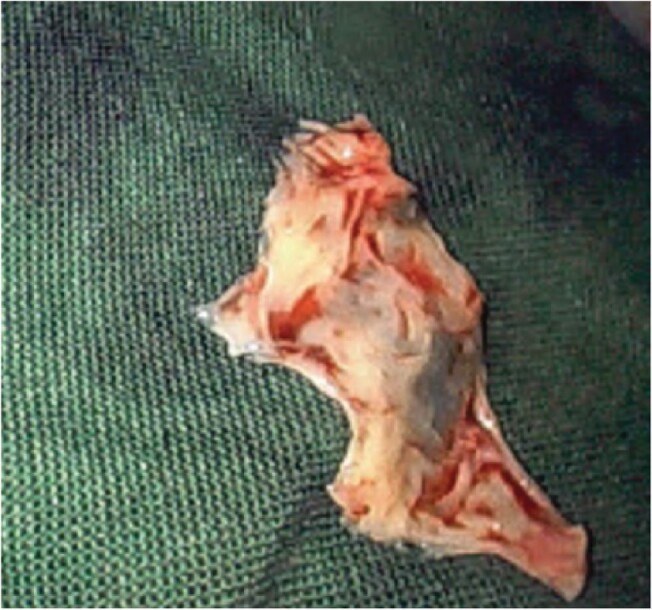
Intraoperative photo of excised redundant mitral valve P2 segment.

On Day 11, after a multidisciplinary team meeting, the patient underwent surgery. The mitral valve was repaired with a triangular resection of the P2 segment and the underlying ruptured chordae (*Figure 3*), reconstruction of the posterior leaflet and implantation of a flexible partial annuloplasty band. The anterior leaflet was freed up to ensure good mobility with a Peeling, Endarterectomy & Decortication (PED) technique. The LVOTO was addressed by an extended septal myectomy performed through a trans-aortic route. Furthermore, a bi-atrial ablative Maze procedure was performed along with right and left atrial appendage closures. Trans-oesophageal echocardiogram after cardiopulmonary bypass demonstrated a mean MV diastolic gradient of 2 mmHg, with no notable LVOT gradient.

Post-operatively, his course was uncomplicated, and the patient was discharged home on Day 16 on bisoprolol 2.5 mg twice daily and frusemide 40 mg daily. Since discharge, he has been progressing well in the community, with return to work one month post-operatively. At six-month follow-up, the patient remained well with a cardiac MRI demonstrating improved parameters.

## Discussion

Assessment of MR in patients with HCM is complex, with a key component being the direction of the MR jet on colour Doppler. Isolated SAM in HCM should typically cause posteriorly directed MR and whenever MR is not in the expected direction, closer mitral valve assessment should be considered.

**Figure 4:**
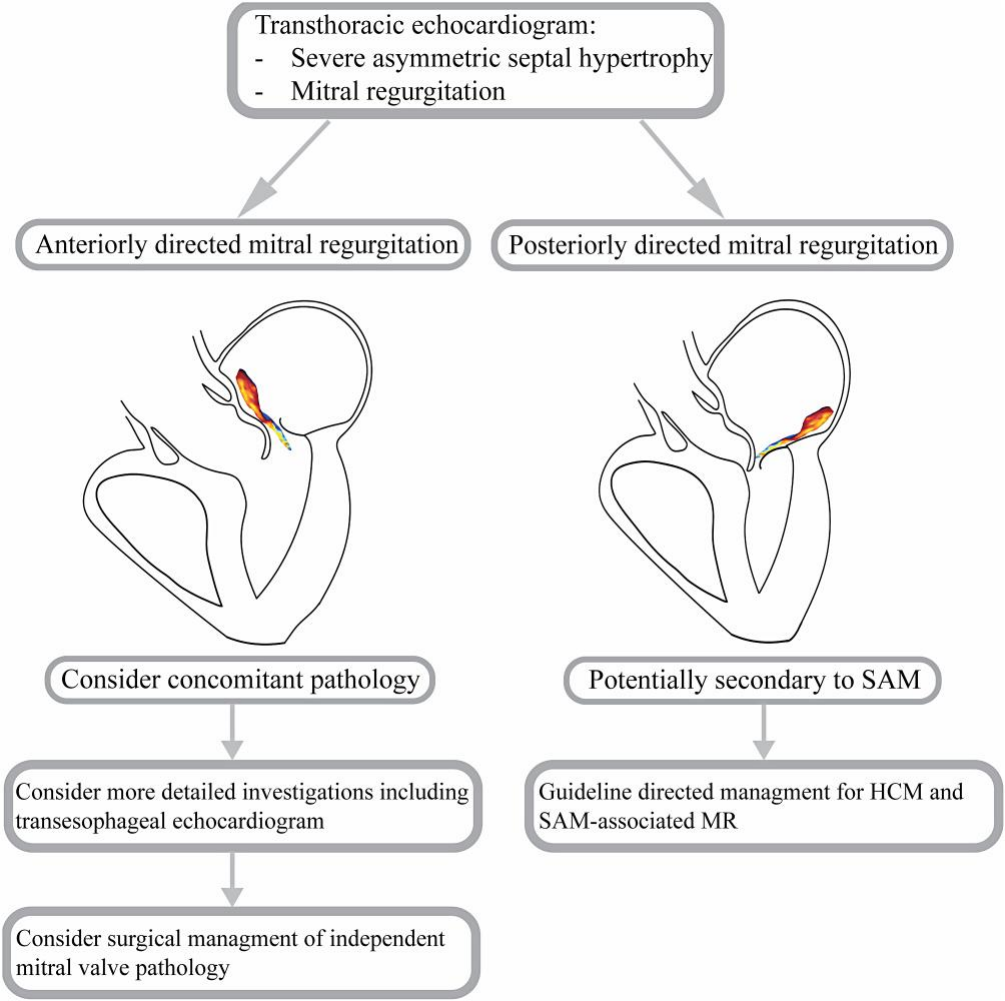
Approach to evaluation of mitral regurgitation in the setting of hypertrophic cardiomyopathy. HCM, hypertrophic cardiomyopathy; SAM, systolic anterior motion; MR, mitral regurgitation.

**Figure 5:**
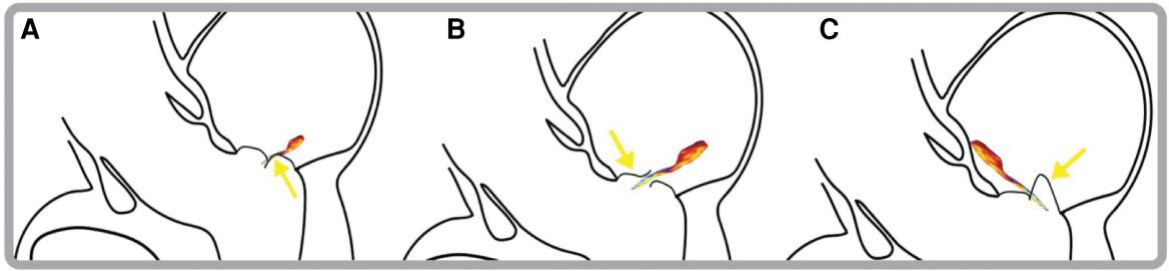
Directions of mitral regurgitation jets. (*A*) MR due to MV restriction (yellow arrow) leading to ipsilateral regurgitation. (*B*) Flail leaflet (yellow arrow) causing contralateral MR as it retracts into the left atrium during systole. (*C*) Prolapse (yellow arrow) of the MV, producing a contralateral MR jet.

Jet direction in MR provides important aetiological and functional information (*Figure 4*). The mechanism of a posteriorly directed jet in SAM-induced MR is due to an anterior and basal movement of the anterior leaflet, resulting in a jet moving posteriorly from the funnel shape formed by the leaflets.^4^ When mitral regurgitation results from restriction of the leaflet apparatus, a MR jet is produced that causes an ipsilaterally directed regurgitation jet (*Figure 5A*).^5^ The impaired ability of the affected leaflet to close causes override by the unaffected leaflet causing backward flow into the atrium on the same side as the dysfunctional leaflet. Conversely, in prolapse, flail, and SAM of the mitral valve, a contralaterally directed MR jet is produced (*Figure 5B* and *C*).^3,5^ As in our case for example, a flail or prolapsing posterior leaflet retracts into the left atrium during systole, resulting in an anteriorly directed regurgitant jet. Systolic anterior motion occurs when the anterior leaflets are drawn into the left ventricular outflow tract during systole, producing a MR jet that flows in the contralateral direction, thus posteriorly directed.

Although the AHA/ACC Guidelines for Hypertrophic Cardiomyopathy caution that anteriorly directed MR may exist in SAM-associated MR, this is rare.^6^ Meanwhile, two-thirds of patients with intrinsic mitral valve disease of the posterior leaflet exhibit central or anteriorly directed jets.^2^ Importantly, it has been suggested that primary mitral valvular disease might be more common in HCM patients compared to the general population. Extended leaflet length, unusual papillary muscle placement, and forward-shifted papillary muscles are all more common in HCM, and mechanisms by which independent mitral valve disease may occur. An analysis of 528 HCM patients identified a 3% rate of independent mitral valve prolapse (as in our patient).^7^ If there is indeed a higher prevalence of primary pathology of the mitral valve apparatus in HCM, then non-posteriorly directed MR jets could be an important red flag to identify on TTE assessment.^6,8^

Our patient’s cardiogenic shock was initially attributed to his HCM and attendant SAM. However, recognition of the significance of the anteriorly directed MR jet (*Figure 1*) prompted further evaluations identifying independent mitral valve pathology.

The 2023 ESC Guidelines for the management of cardiomyopathies specify the importance of carefully evaluating MV structure and function prior to invasive septal reduction procedures.^9^ Our case offers an example of the clinical relevance and potential use of jet direction as a screening tool for consideration of further detailed mitral valve assessment prior to cardiac surgery. Future use of myosin inhibitors (such as mavacamten) may offer novel methods of non-surgically reducing LVOTO to better define mitral anatomy and relative contributions of competing pathologies.

## Conclusion

Isolated SAM in HCM should classically cause posteriorly directed MR. If non-posteriorly directed MR is present, this should alert the clinician for concomitant mitral valve pathology and prompt detailed investigations.

## Lead author biography


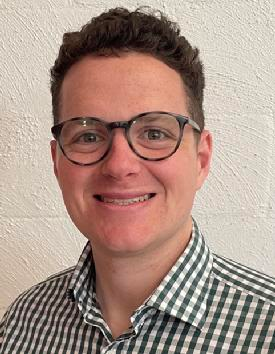
Vincenzo Somma is currently completing basic physician training at St Vincent’s Hospital Melbourne in Victoria, Australia.

## Supplementary material

Supplementary material is available at *European Heart Journal – Case Reports* online.

**Consent:** The patient provided informed consent for their case to be published in a deidentified form, compliant with COPE guidelines.

**Funding:** E.P. is supported by the Wilma Beswick Senior Research Fellowship at Melbourne University, the National Heart Foundation, Viertel Fellowship and the Mamoma Foundation.

## Supplementary Material

ytae121_Supplementary_Data

## Data Availability

Deidentified data are available upon reasonable request.
